# Laser-Induced Forward Transferred Optical Scattering Nanosilica for Transparent Displays

**DOI:** 10.3390/nano12203674

**Published:** 2022-10-19

**Authors:** Ruo-Zhou Li, Mingqing Yang, Lvjiu Guo, Ke Qu, Tong Jian, Ying Yu, Jing Yan

**Affiliations:** 1 College of Integrated Circuit Science and Engineering, Nanjing University of Posts and Telecommunications, Nanjing 210023, China; 2 National and Local Joint Engineering Laboratory of RF Integration and Micro Assembly Technology, Nanjing University of Posts and Telecommunications, Nanjing 210023, China; 3 College of Electronic and Optical Engineering, Nanjing University of Posts and Telecommunications, Nanjing 210023, China

**Keywords:** laser-induced forward transfer, transparent displays, scattering, silica nanoparticle, laser printing

## Abstract

Laser printing has become a promising alternative for large-scale fabrication of functional devices. Here, laser-induced forward transfer (LIFT) of nanosilica was successfully achieved using a lower-cost nanosecond laser with a center wavelength of 1064 nm. To enhance the light absorption of silica, a small amount of graphene oxide (GO) was added to the fumed silica. Investigations were conducted to give an insight into the role of GO in the LIFT process. Pattern deposition was achieved with a minimum line width of 221 μm. The scattering can be tuned from ~2.5% to ~17.5% by changing the laser fluence. The patternable transparent display based on laser transferred nanosilica (LTNS) film was also demonstrated, showing its capability to deliver information on multiple levels. This LIFT based technique promotes fast, flexible, and low-cost manufacturing of scattering-based translucent screens or patterns for transparent displays.

## 1. Introduction

**Figure 1:**
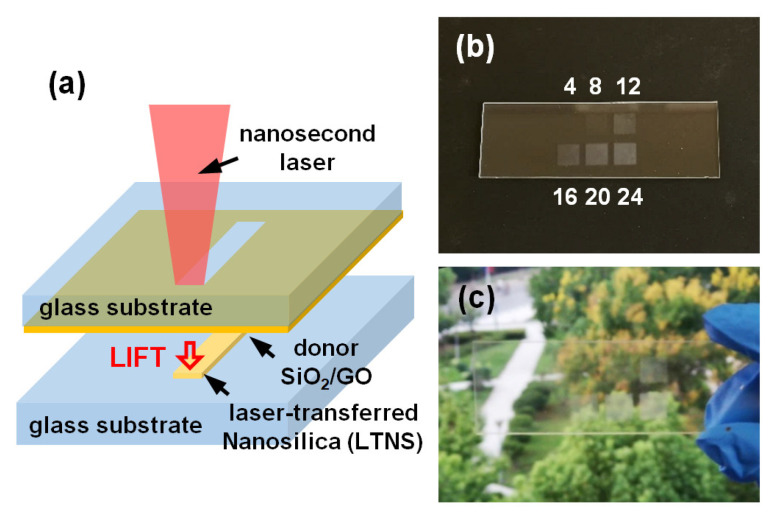
(**a**) Schematic diagram of the processing system; (**b**,**c**) show the photographs of laser-transferred nanosilica (LTNS).

Laser printing has become a promising alternative for large-scale fabrication of electronic and optical devices in an on-demand, non-contact, and highly controllable way [1,2]. A focused laser beam can modify the surface features or deposit materials through mechanisms such as laser-induced ablation [3,4], sintering [5], reduction [6], carbonization [7], polymerization [8], metallization [9,10], etc. Among them, laser-induced forward transfer (LIFT) uses transfer mechanism to deposit materials onto target samples. A LIFT setup typically consists of a thin donor layer previously deposited onto a transparent carrier, and a receiver substrate placed closely to the donor layer (Figure 1a). The laser pulse irradiates the interface between the donor layer and the transparent carrier, leading to the ejection of the donor material towards the receiver substrate [11]. This single-step, non-destructive, and additive nature of deposition is compatible with a wide number of potential materials without the concern of the drawbacks such as nozzle clogging in inject printing, thereby attracting growing interest for various potential applications in both electrical and optical applications [1,12,13].

Nanosilica can scatter incident light for numerous applications such as diffusion optics, lighting, displays, and solar cells. In particular, nanosilica can be applied to scattering-based transparent displays [14,15,16]. This is because a scattering-based transparent display often relies on a transparent screen embedded with nanomaterials to scatter the incident light from a projector [17]. Therefore, a person views information on the screen and can see the real-world content behind the screen simultaneously. Many attractive applications can be realized through this ‘see-through’ function: navigating information can be displayed on car windshields to enhance the driving experience [16,18]; eyeglasses can become monitors for human-machine interaction and entertainment [19]; advertisements and product information can be displayed on show windows for shopping [16]. Extensive studies focus on the synthesis and structural design of silica-based scattering materials for transparent display. Nevertheless, the fabrication of nanosilica on the transparent screen is mainly limited to drop casting and rod coating [14,15,16], much less pattern deposition. It would be more advantageous to induce LIFT to print transparent and scattering nanosilica films for transparent display. 

Light-matter interactions in a LIFT process rely on the light absorption in the laser wavelength. However, the light absorption for silica is tiny in the visible and near infrared (NIR) bands, resulting in difficulty in transferring silica material onto a receiver substrate. The LIFT fabrication of transparent (low absorption) materials typically uses an absorbing layer which insets into the interface between the donor material and carrier substrate to enhance light absorption [20]. Laser irradiation on the absorbing layer generates gases that actuate the donor material fragments towards the receiver substrate [1]. Ultraviolet (UV) nanosecond lasers were also used for SiO_x_ materials [21,22] because the absorption coefficient of SiO_x_ is relatively higher in the UV region than in the visible to NIR band. Multi-photon absorption of femtosecond pulses can also promote the LIFT fabrication of transparent silica donors [23]. 

Herein, we first report LIFT fabrication of nanosilica for transparent displays using a lower-cost nanosecond laser with a center wavelength of 1064 nm. Since silica has insufficient light absorption, a small amount of graphene oxide (GO) was added to silica. Investigations were conducted to give an insight into the role of GO in the LIFT process. Pattern deposition was achieved and illustrated. The scattering can be tuned by changing the laser fluence. The patternable transparent display based on laser transferred nanosilica (LTNS) film was also demonstrated, showing the capability of delivering information at multiple levels. This LIFT based technique promotes fast, flexible, and low-cost manufacturing of scattering-based translucent screen or patterns for transparent display.

## 2. Materials and Methods

Fumed silica (AEROSIL R 972) after-treated with dimethyldichlorosilane (DDS) was purchased from Evonik Industries AG, Essen, Germany. Ethanol was purchased from Shanghai Aladdin Biochemical Technology Co., Ltd, Shanghai, China. All reagents were used without further purification. 

The donor film was prepared by spin-coating of silica/GO ethanol dispersion onto a microscope glass slide. Specifically, GO was synthesized from graphite flakes via a modified Hummers method which has been described elsewhere [24,25] and the morphology can be seen from the SEM image in Figure S1a. Then, 21 mg GO and 100 mg fumed silica were added to 5 mL ethanol followed by stirring and ultrasonication for 10 min. The ratio of the GO: SiO_2_ mixture was chosen taking note of the absorption of the donor film, as a higher absorption can reduce the transfer threshold. Another issue is cost-cutting, because GO (~$1/g) is more expensive than fumed silica (<$0.05/g). The mixture was spin-coated on to the glass slide with a speed of 300 rpm for 30 s and then baked on a hotplate at 50 °C for 5 min. The 16 nm-sized fumed silica nanoparticles formed a donor film of 4 μm in average thickness as shown in Figure S1b. Plenty of aggregates were spotted on the donor film as the SEM images show in Figure S1c,d. 

The LIFT was conducted at ambient air environment and room temperature. Figure 1a shows the schematic diagram of the LIFT system [12]. The nanosecond fiber laser (JPT YDFLP, Shenzhen JPT Opto-electronics Co., Ltd., Shenzhen, China) with a wavelength of 1064 nm was used for sample fabrication with a pulse duration of 10 ns and a repetition rate of 45 kHz. The laser beam was focused on the interface between a donor film and a carrier glass slide by an f-theta lens (Excelitas Technologies, Waltham, MA, USA) with a focal length of 100 mm and a spot size of 30 μm. A scanning galvo mirror (Sino-Galvo (Jiangsu) Technology Co., Ltd., Zhenjiang, China) was used for laser beam scanning at a speed of 1 m/s. A bidirectional scanning strategy was used with a line spacing (hatch distance) of 50 μm and a pulse interval of 22.2 μm as shown in Figure S2. The laser fluencies were set at 4 to 24 J/cm^2^, corresponding to pulse energies of 45 to 270 μJ. The distance between the donor and the receiver glass slide was 50 μm. 

The scanning electron microscope (SEM) observations combined with energy dispersive spectroscopy (EDS) were obtained using a Siama300 (Carl Zeiss AG, Jena, Germany) and an Xplore30 EDS detector (Oxford Instruments, Abingdon, United Kingdom), respectively. Microscope images were acquired from an optical microscope (BX53M, Olympus Corporation, Tokyo, Japan). The X-ray photoelectron spectroscopy (XPS) patterns were obtained by a Thermo K-alpha spectrometer (Thermo Fisher Scientific Inc., Waltham, MA, USA) with a monochromatic Al-Ka (1486.6 eV) X-ray source. The pass energy was 20 eV for the high-resolution scan. Raman spectra were measured using an HR Evolution spectrometer (HORIBA, Ltd., Kyoto, Japan). The VIS-NIR spectra (transmission and forward scattering) were obtained by a TE-cooled spectrometer (SM303, Spectral Products, Putnam, CT, USA) equipped with an integrating sphere (2P4, Thorlabs, Inc., Newton, NJ, USA) as shown in Figure S3. A lab-built projection system was used to project images onto the laser-printed screen. 

## 3. Results and Discussion

### 3.1. GO-Promoted Laser Trasnfer of Nanosilica

It should be mentioned that pure fumed silica donor film without GO cannot be transferred even at the highest laser fluence (40 J/cm^2^) that the laser can provide. To lower the transfer threshold, a small amount of GO was added to the silica dispersion in the weight proportion of 1: 5. Laser transfer was then conducted with some typical laser fluence values ranging from 4 to 24 J/cm^2^. In the presence of GO, the transfer started to occur at a significantly lower laser fluence of 8 J/cm^2^, leading to slightly matt white colors on the receiver glass substrate as shown in Figure 1b. The laser transferred nanosilica (LTNS) was also semitransparent (Figure 1c), so that the view behind the sample can be seen through LTNS.

**Figure 2:**
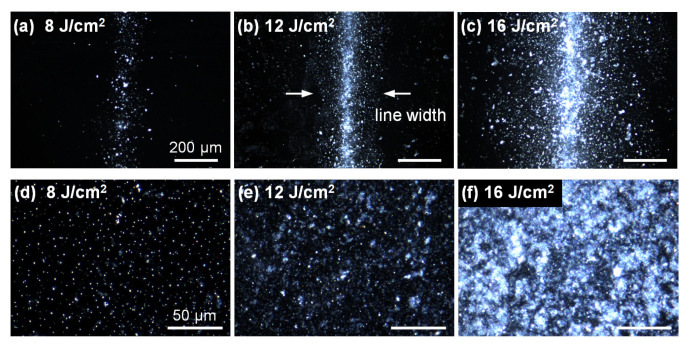
Dark field microscope images of the LTNS lines obtained at (**a**) 8 J/cm^2^, (**b**) 12 J/cm^2^, and (**c**) 16 J/cm^2^. The white arrows in (**b**) indicates line width. LTNS films obtained at (**d**) 8 J/cm^2^, (**e**) 12 J/cm^2^, and (**f**) 16 J/cm^2^. Scale bars in figures (**b**,**c**) and (**e**,**f**) indicate 200 μm and 500 μm, respectively.

Figure 2a–c show the dark field microscope images of the LTNS in line printing cases with several laser fluencies. The white specks were attributed to the light scattering by the LTNS. Some splashes and debris appeared at the flanks of the printed line, indicating that the donor material was ejected into the surrounding area. Film printing results are illustrated in Figure 2d–f, displaying a prominently increased light scattering intensity with higher laser fluence from 8 to 16 J/cm^2^ and a slight drop in the intensity at 24 J/cm^2^ (Figure S4).

**Figure 3:**
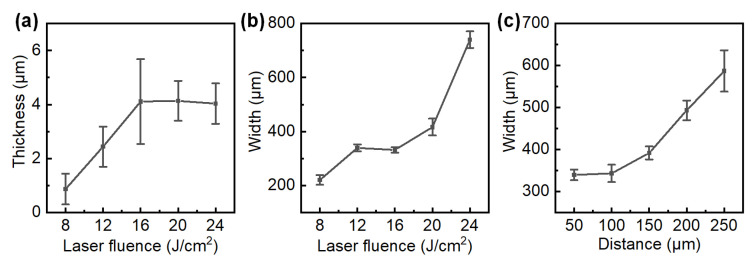
(**a**) Film thickness, and (**b**) line width of LTNS as a function of laser fluence. (**c**) Line width as a function of transfer distance between the donor film and receiver substrate.

The average thickness of the film increases to a maximum value of about 4 μm at a laser fluence of 16 J/cm^2^, and the value is maintained when further increasing the laser fluence as shown in Figure 3a. This trend is consistent with the dark field results because a larger amount of transferred silica scatters lights more strongly, generating brighter images. The line widths, which indicate spatial resolution, ramp up with both the increasing laser fluence (Figure 3b) and the transfer distance between the carrier and receiver substrates (Figure 3c). For a 50 μm transfer distance, the line width has a minimum value of 221 μm at 8 J/cm^2^, and gradually widens to 740 μm at 24 J/cm^2^. The line width may be further reduced by reducing the laser spot size or using a femtosecond laser. Note that the typical pixel size for a commercial liquid crystal display (LCD) monitor (1080 p, 27 inch) is about 300 μm, and is even larger (1.25 to 10.00 mm) for outdoor light-emitting diode (LED) displays. Therefore, the spatial resolutions of the proposed LTNSs fulfill many applications of transparent displays, such as head-up display (HUD), show windows, or outdoor advertising.

**Figure 4:**
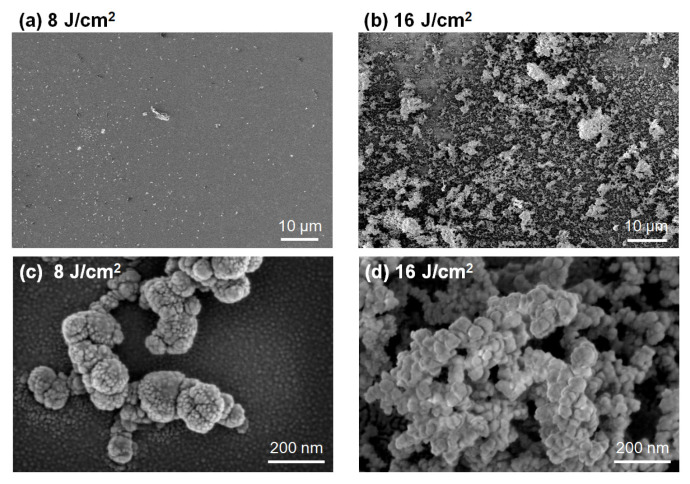
SEM images of the LTNS film obtained at (**a**) 8 J/cm^2^ and (**b**) 16 J/cm^2^. (**c**,**d**) show higher magnification images.

The SEM images illustrate some typical morphologies of the LTNS corresponding to 8, 12 and 16 J/cm^2^. The 8 J/cm^2^ case (Figure 4a) induces uniform-distributed fragments on the substrate surface. The fragments consisted of silica grains, as shown in Figure 4c. The average diameter of the grains was approximately 16 nm which was consistent with that of the fumed silica (AEROSIL R 972). For the 16 J/cm^2^ case (Figure 4b,d), much-larger-sized fragments splashed over the substrate with high surface coverage and abundant porosity. The average grain size was increased to 43 μm. This may be due to the melting of the silica nanoparticles at higher laser fluence because laser irradiation can generate intensive heat and the small size effects can also lower the melting temperature of the nanoparticles [1,25]. The results (Figure S5) corresponding to 24 J/cm^2^ are similar to those obtained at 16 J/cm^2^. 

**Figure 5:**
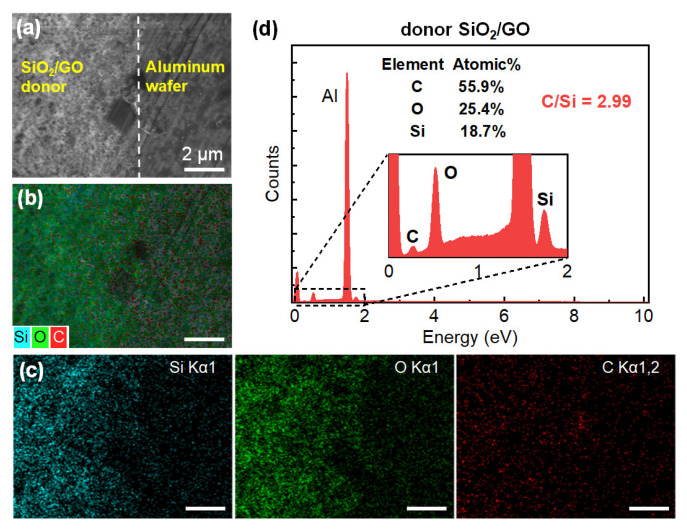
(**a**) SEM image, (**b**,**c**) elemental mapping analysis, and (**d**) EDX analysis of the donor film. The white dash line roughly indicates the boundary between the donor film and bare Al wafer. The scale bars in figures (**b**,**c**) indicate 2 μm.

**Figure 6:**
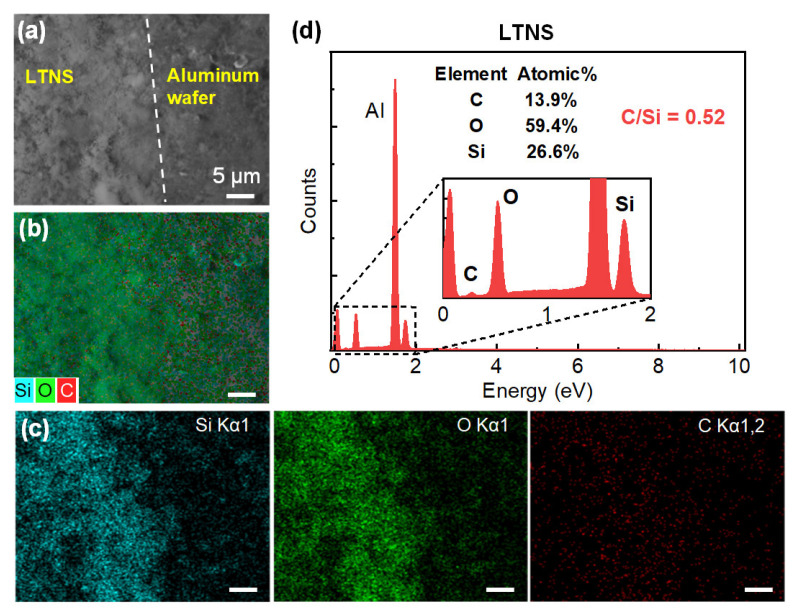
(**a**) SEM image, (**b**,**c**) elemental mapping analysis, and (**d**) EDX analysis of the LTNS film. The white dash line roughly indicates the boundary between the LTNS film and bare Al wafer. The scale bars in figures (**b**,**c**) indicate 5 μm.

By taking EDS elemental mapping, the transfer of the LTNS was further confirmed. The observations were obtained from the top of the sample surfaces at the donor film/Al wafer boundary (Figure 5) and the LTNS film/Al wafer boundary (Figure 6). The boundaries were fuzzy, with the amount of material decreasing gradually from the film to the wafer side. Some fragments are also distributed in the Al wafer area. This is similar to the sample on glass wafer which has been illustrated in the microscope images in Figure 2b. The colors gradually fade out across the boundary from the donor or LTNS film to the Al wafer, clearly implying the presence of carbon, silicon, and oxygen elements. These elements were corresponding to and SiO_2_ and GO. Note that, the LTNS film shows a significant drop in the elemental proportion of carbon from 55.9% to 13.9%. The C/Si ratios were also reduced from 2.99 to 0.52 for the donor and LTNS, respectively. Therefore, the proportion of GO was reduced.

**Figure 7:**
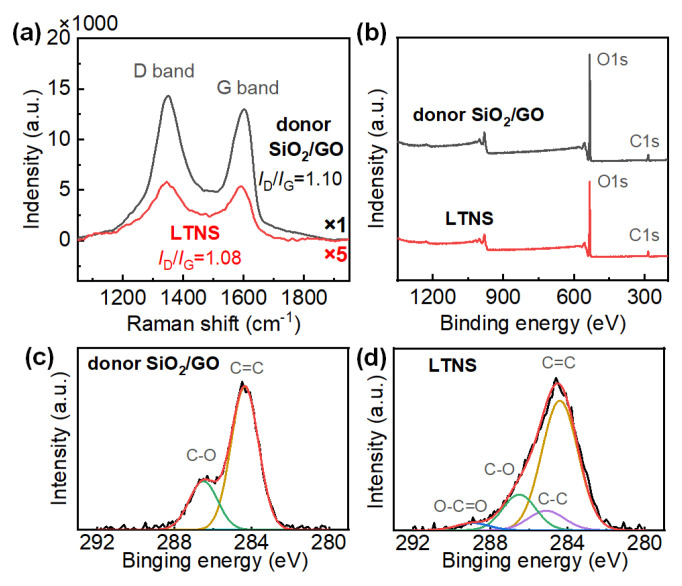
(**a**) Raman spectra and (**b**) XPS spectra of the donor film and the LTNS film. The C1s peaks for (**c**) the donor film, and (**d**) the LTNS film.

The decrease of GO proportion was also seen from the Raman spectra as shown in Figure 7a. The G band results from E_2g_ phonon scattering of graphitic structure and the D band is a defect band which can be attributed to the dangling bands in plane terminations [26]. Compared with the donor film, the LTNS film displayed remarkably lower peak intensities by over one order of magnitude. 

Surface chemistry of both donor film and LTNS was further analyzed by XPS, as shown in Figure 7b. The C1s spectra are shown in Figure 7c,d for donor film and LTNS film, respectively. For the donor film, two Gaussian peaks with binding energies of 284.4 and 286.5 eV are attributed to C=C and O-C=O bonds in GO, respectively [27]. However, compared with the donor film, the LTNS film shows fewer C-O bonds. Two weak peaks corresponding to C-C (285.1 eV), and O-C=O (288.9 eV) bonds were also observed. The factors above imply the oxygen-containing C-O function groups have been removed partially from GO [28]. Therefore, C-C bonds increased. Decomposition of GO may also have occurred when the GO was exposed to the laser.

In general, GO promoted the laser transfer of nanosilica in two aspects. Firstly, GO enhances the light absorption of the donor at 1064 nm. Adding a small amount of GO to the fumed silica with a weight ratio of 1:5 can double the absorption from 7.7% to 16.9%, as shown in Figure S6. This leads to a stronger light-matter interaction that promotes the laser transfer. Secondly, laser irradiation caused local heat and intensive temperature rise, which may lead to oxidative burning of GO to volatile gases [29]. This was evidenced by the EDS, Raman and XPS as the proportion of GO was significantly reduced and the functional groups of GO were modified. The released gases can actuate and accelerate the donor material outward from the carrier substrate [30]. Consequently, only the donor films with GO were transferred at the low laser fluence of 8 J/cm^2^; the transfer did not occur for the film without GO even at a significantly higher laser fluence of 40 J/cm^2^.

### 3.2. Nanosilica-Induced Light Scattering for Transparent Displays

**Figure 8:**
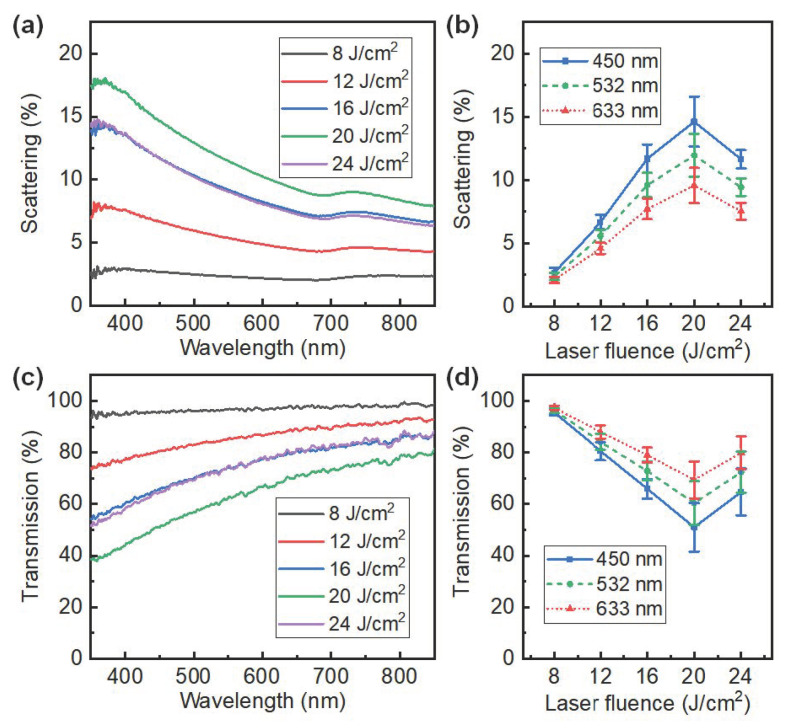
Optical properties of LTNS films. (**a**) Forward scattering spectra and (**c**) transmission spectra with a set of laser fluencies. (**b**) Forward scattering coefficient and (**d**) transmission coefficient as a function of laser fluencies at wavelengths of 450, 532, and 633 nm.

Light scattering and transmission are critical for nanosilica-enabled transparent displays. The optical spectra of the LTNS films were measured at some laser fluencies. Figure 8a plots the forward scattering spectra of the LTNS. The stronger scattering at shorter wavelength regions can be attributed to Mie scattering. This is because the feature size in LTNS is similar to or larger than the wavelength of the incident light, and the scattering strength is negatively correlated with the wavelength. The scattering versus the laser fluence at three wavelengths of 450 nm (blue), 532 nm (green) and 633 nm (red) is displayed in Figure 8b. The scattering was about 2.5% at 8 J/cm^2^, and reached a maximum value of about 8%, 12%, and 14% at 20 J/cm^2^ for the three wavelengths of 450 nm, 532 nm, and 633 nm, respectively. The transmission spectra in Figure 8c,d show the opposite tendency to the scattering spectra. The transmission values can be tuned between 40% and 96% (Figure 8d). The scattering transparent display presented here requires both a high transmission to see-through and an appropriate scattering for efficiency concerns. Considering the trade-off between scattering and transmission, a laser fluence of 16 J/cm^2^ was chosen for the demonstrations, with a corresponding transmission of 60% to 82% and scattering of 8% to 14% in the visible band. 

**Figure 9:**
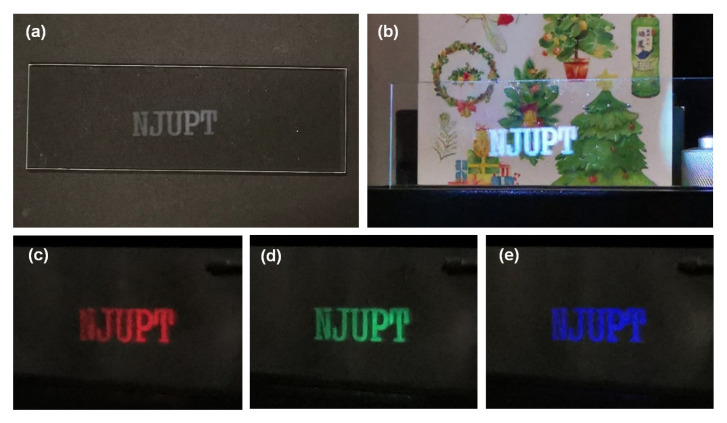
(**a**) Photograph of the laser-printed letters NJUPT on a glass slide. (**b**–**e**) Letters were light up by white, red, green, and blue light.

Patternable transparent display was first demonstrated with laser-printed letters NJUPT on a glass slide as shown in Figure 9a. When white light illuminated the whole area of the glass slide, only the letters were lighted up (Figure 9b) due to light scattering. The blank areas without LTNS remained transparent, showing the background view. The color of the letters can be tuned according to the color of the incident light as illustrated in Figure 9c–e.

**Figure 10:**
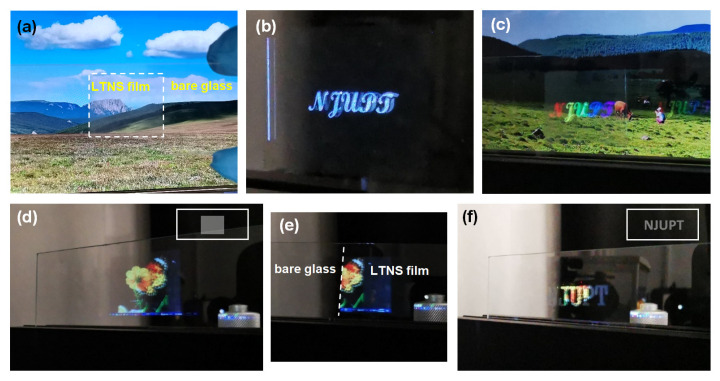
(**a**) Photograph of the laser-printed LTNS film on a glass slide. (**b**–**e**) Letters and images were projected on to the LTNS film. (**f**) The image was projected onto the letters NJUPT.

The LTNS film can be see-through as a translucent screen with a decent visibility of the view behind it (Figure 10a). Figure 10b,c show the white and colorful letters projected on the film, respectively. Further demonstrations of image display are presented in Figure 10d. A photo image of a butterfly on a flower was clearly projected on the screen. Figure 10e compares the difference in effect with and without the LTNS on the glass slide. The image appeared only at the locations with LTNS, proving the role of the LTNS film. The image was also projected on the LTNS pattern (letters NJUPT) illustrated in Figure 10f. Therefore, the patternable LTNS film can deliver information on multiple levels.

## 4. Conclusions

LIFT fabrication of nanosilica was successfully achieved using a lower-cost nanosecond laser with a center wavelength of 1064 nm. By adding a small amount of GO to the fumed silica, the laser transfer can be triggered at a laser fluence above 8 J/cm^2^. Two aspects of GO may be involved in the LIFT process, the enhancement of light absorption and gas generation due to laser-induced decomposition and reduction. The LIFT process enables pattern deposition with a typical minimum line width of 221 μm. The scattering can be tuned from ~2.5% to ~17.5% by changing the laser fluence. The patternable transparent display based on LTNS film was also demonstrated, showing its ability to deliver information on multiple levels. This LIFT based technique promotes fast, flexible, and low-cost manufacturing of scattering-based translucent screens or patterns for transparent displays.

## Data Availability

The data presented in this paper are available on reasonable request from the corresponding author.

## Supplementary Materials

The following supporting information can be downloaded at: www.mdpi.com/article/10.3390/nano12203674/s1. Figure S1: SEM image of (a) graphene oxide. (b) Cross-sectional microscope image of the donor film. (c) SEM image of the donor film, and (d) an enlarged view. Figure S2: Schematic diagram of the laser bidirectional scanning strategy. Figure S3: Optical setup of the transmission measurement and forward scattering measurement with an integrating sphere in (a) transmission and (b) forward scattering setup. Figure S4: Dark field microscope images of the LTNS films obtained at (a) 20 J/cm^2^ and (b) 24 J/cm^2^. Figure S5: (a) SEM images of the LTNS film obtained at 24 J/cm^2^, and (b) the enlarged view. Figure S6: NIR absorption spectra of the donor films.

## References

[1] Hu A. (2020). Laser Micro-Nano-Manufacturing and 3D Microprinting.

[2] Pfleging W. (2020). Recent progress in laser texturing of battery materials: A review of tuning electrochemical performances, related material development, and prospects for large-scale manufacturing. Int. J. Extrem. Manuf..

[3] Mishra S., Yadava V. (2015). Laser Beam MicroMachining (LBMM)—A review. Opt. Laser Eng..

[4] Chen T.H., Fardel R., Arnold C.B. (2018). Ultrafast z-scanning for high-efficiency laser micro-machining. Light Sci. Appl..

[5] Li R.-Z., Ji J., Liu L., Wu Z., Ding D., Yin X., Yu Y., Yan J. (2022). A high-sensitive microwave humidity sensor based on one-step laser direct writing of dielectric silver nanoplates. Sens. Actuators B Chem..

[6] Li R.-Z., Wu Z., Ji J., Yin X., Yan J., Fang Y., Yu Y. (2021). A Wideband Termination Based on Laser-Scribed Lossy Microstrip Line Structures. Appl. Sci..

[7] Cheng C., Wang S., Wu J., Yu Y., Li R., Eda S., Chen J., Feng G., Lawrie B., Hu A. (2016). Bisphenol A Sensors on Polyimide Fabricated by Laser Direct Writing for Onsite River Water Monitoring at Attomolar Concentration. ACS Appl. Mater. Interfaces.

[8] Li Y., Fullager D.B., Angelbello E., Childers D., Boreman G., Hofmann T. (2018). Broadband near-infrared antireflection coatings fabricated by three-dimensional direct laser writing. Opt. Lett..

[9] Li R.-Z., Yan J., Fang Y., Fan X., Sheng L., Ding D., Yin X., Yu Y. (2019). Laser-Scribed Lossy Microstrip Lines for Radio Frequency Applications. Appl. Sci..

[10] Bai S., Serien D., Ma Y., Obata K., Sugioka K. (2020). Attomolar Sensing Based on Liquid Interface-Assisted Surface-Enhanced Raman Scattering in Microfluidic Chip by Femtosecond Laser Processing. ACS Appl. Mater. Interfaces.

[11] Delaporte P., Alloncle A.-P. (2016). [INVITED] Laser-induced forward transfer: A high resolution additive manufacturing technology. Opt. Laser Technol..

[12] Li R.-Z., Guo L., Liu L., Yang M., Fang Y., Yu Y., Yan J. (2022). Laser-Induced Forward Transfer of Silver Nanoparticles for a Black Metal Absorber. Front. Phys..

[13] Zywietz U., Evlyukhin A.B., Reinhardt C., Chichkov B.N. (2014). Laser printing of silicon nanoparticles with resonant optical electric and magnetic responses. Nat. Commun..

[14] Seyyedi M., Rostami A., Matloub S. (2020). Comparative study of transparent display using aperiodic arrays of Si–SiO_2_ core–shell nanoparticles. Opt. Quant. Electron..

[15] Son I., Lee J.H. (2020). Highly Transparent and Wide Viewing Optical Films Using Embedded Hierarchical Double-Shell Layered Nanoparticles with Gradient Refractive Index Surface. ACS Appl. Mater. Interfaces.

[16] Dolatyari M., Jafari A., Rostami A., Klein A. (2019). Transparent Display using a quasi-array of Si-SiO_2_ Core-Shell Nanoparticles. Sci. Rep..

[17] Yan J., Fan X., Liu Y., Yu Y., Fang Y., Li R.-Z. (2022). Passive patterned polymer dispersed liquid crystal transparent display. Chin. Opt. Lett..

[18] Hsu C.W., Zhen B., Qiu W., Shapira O., DeLacy B.G., Joannopoulos J.D., Soljacic M. (2014). Transparent displays enabled by resonant nanoparticle scattering. Nat. Commun..

[19] Monti A., Toscano A., Bilotti F. (2017). Analysis of the scattering and absorption properties of ellipsoidal nanoparticle arrays for the design of full-color transparent screens. J. Appl. Phys..

[20] Papazoglou S., Zergioti I. (2017). Laser Induced Forward Transfer (LIFT) of nano-micro patterns for sensor applications. Microelectron. Eng..

[21] Ihlemann J., Weichenhain-Schriever R. (2014). Patterned deposition of thin SiOX-films by laser induced forward transfer. Thin Solid Films.

[22] Ihlemann J., Weichenhain-Schriever R. (2010). Laser-induced congruent forward transfer of SiOx -layers. Appl. Phys. A.

[23] Banks D.P., Kaur K.S., Eason R.W. (2009). Etching and forward transfer of fused silica in solid-phase by femtosecond laser-induced solid etching (LISE). Appl. Surf. Sci..

[24] Wang H., Robinson J.T., Li X., Dai H. (2009). Solvothermal reduction of chemically exfoliated graphene sheets. J. Am. Chem. Soc..

[25] Li R.-Z., Peng R., Kihm K., Bai S., Bridges D., Tumuluri U., Wu Z., Zhang T., Compagnini G., Feng Z. (2016). High-rate in-plane micro-supercapacitors scribed onto photo paper using in situ femtolaser-reduced graphene oxide/Au nanoparticle microelectrodes. Energy Environ. Sci..

[26] Zhang Y., Guo L., Wei S., He Y., Xia H., Chen Q., Sun H.-B., Xiao F.-S. (2010). Direct imprinting of microcircuits on graphene oxides film by femtosecond laser reduction. Nano Today.

[27] Shown I., Hsu H.C., Chang Y.C., Lin C.H., Roy P.K., Ganguly A., Wang C.H., Chang J.K., Wu C.I., Chen L.C. (2014). Highly efficient visible light photocatalytic reduction of CO_2_ to hydrocarbon fuels by Cu-nanoparticle decorated graphene oxide. Nano Lett..

[28] Arul R., Oosterbeek R.N., Robertson J., Xu G., Jin J., Simpson M.C. (2016). The mechanism of direct laser writing of graphene features into graphene oxide films involves photoreduction and thermally assisted structural rearrangement. Carbon.

[29] Zhou Y., Bao Q., Varghese B., Tang L.A., Tan C.K., Sow C.H., Loh K.P. (2010). Microstructuring of graphene oxide nanosheets using direct laser writing. Adv. Mater..

[30] Fardel R., Nagel M., Nuesch F., Lippert T., Wokaun A. (2010). Laser-induced forward transfer of organic LED building blocks studied by time-resolved shadowgraphy. J. Phys. Chem. C.

