# Supplementary material for: Laser-Induced Forward Transferred Optical Scattering Nanosilica for Transparent Displays
Source: Nanomaterials (Basel). 2022 Oct 19;12(20):3674. doi: 10.3390/nano12203674 (PMC9610747; doi:10.3390/nano12203674)
Supplement: Supplementary file 1 [file nanomaterials-12-03674-s001.zip › nanomaterials-1974346-supplementary.pdf]

nanomaterials

MDPI

Supporting Information

Laser-Induced Forward Transferred Optical Scattering Nanosilica for Transparent Displays

Ruo-Zhou Li [1,2], Mingqing Yang [1,2], Lvjiu Guo [1], Ke Qu [3], Tong Jian [3], Ying Yu [1,2] and Jing Yan [3,*]

[1] College of Integrated Circuit Science and Engineering, Nanjing University of Posts and Telecommunications, Nanjing 210023, China
[2] National and Local Joint Engineering Laboratory of RF Integration and Micro Assembly Technology, Nanjing University of Posts and Telecommunications, Nanjing 210023, China
[3] College of Electronic and Optical Engineering, Nanjing University of Posts and Telecommunications, Nanjing 210023, China
* Correspondence: jing.yan@njupt.edu.cn

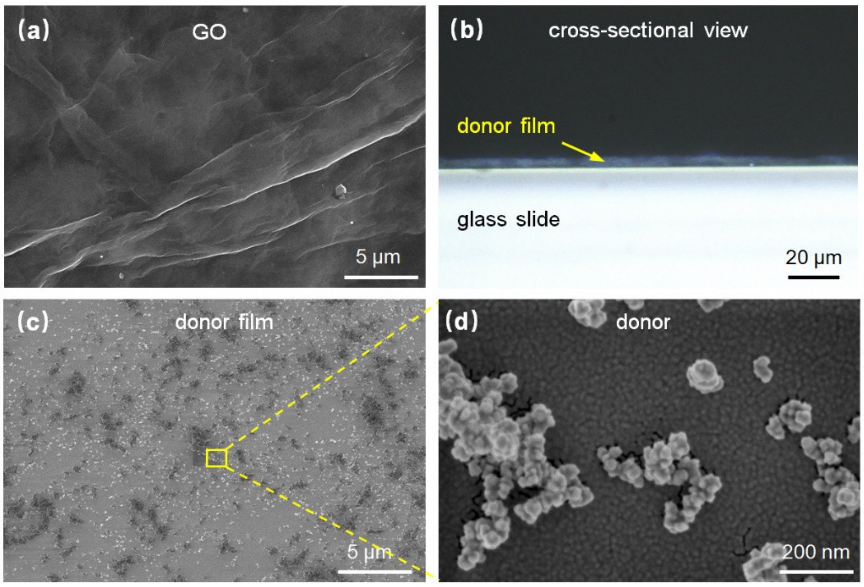

Figure S1. SEM image of (**a**) graphene oxide. (**b**) Cross-sectional microscope image of the donor film. (**c**) SEM image of the donor film, and (**d**) an enlarged view.

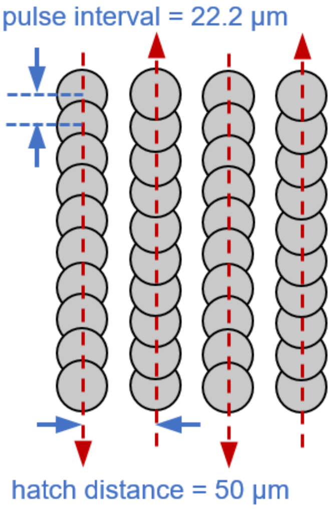

Figure S2. Schematic diagram of the laser bidirectional scanning strategy.

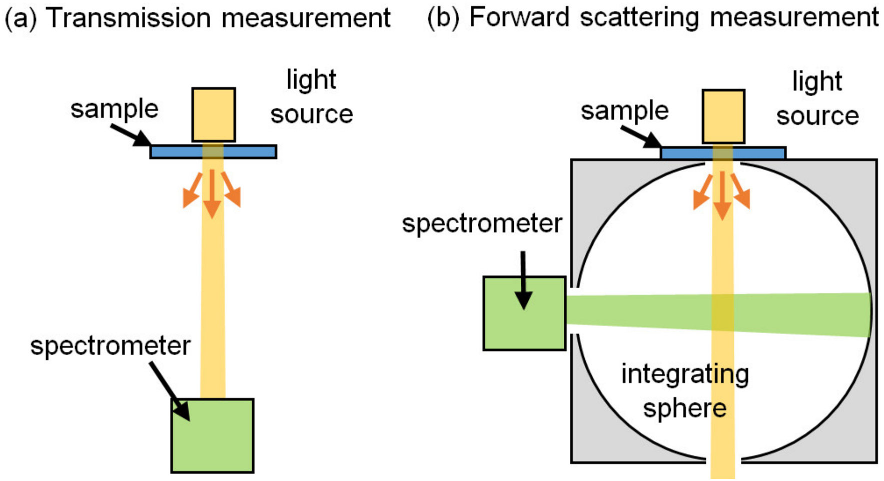

Figure S3. Optical setup of the transmission measurement and forward scattering measurement with an integrating sphere in (**a**) transmission and (b) forward scattering setup.

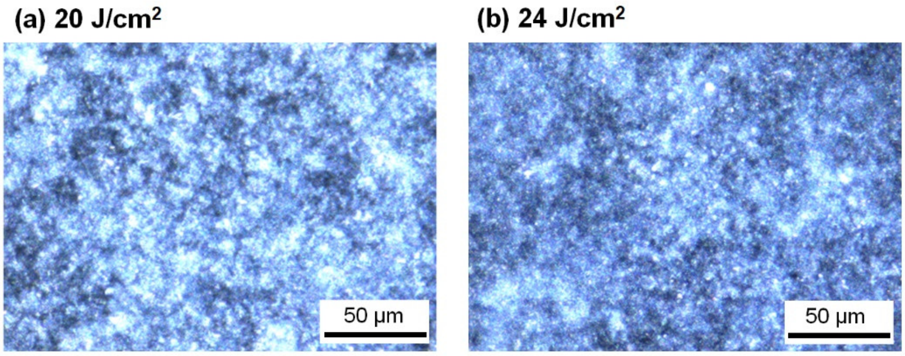

Figure S4. Dark field microscope images of the LTNS films obtained at (a) 20 J/cm^2 and (**b**) 24 J/cm^2.

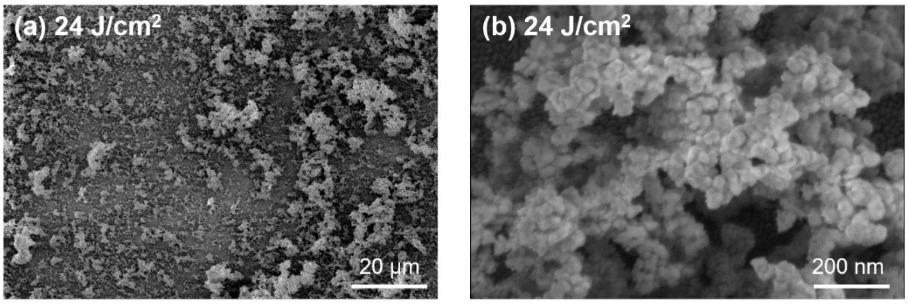

Figure S5. (**a**) SEM images of the LTNS film obtained at 24 J/cm^2, and (**b**) the enlarged view.

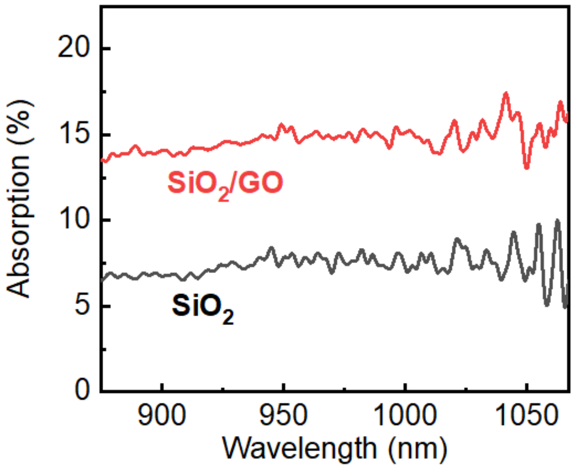

Figure S6. NIR absorption spectra of the donor films.
